# Foreign

**Published:** 1841-01-16

**Authors:** 


### FOREIGN.

*Statical Researches on Pneumonia.*—The following conclusions are deduced from a careful comparison of seventy-five cases of active pneumonia observed in the wards of La Charité hospital, and under the care of M. Bouillaud. They have been collected by M. Pelletan; and his memoir, founded upon them, was recently reported upon to the Royal Academy by M. Rayer to whom it had been referred.

1. Pneumonia affecting one lung is more frequent than pneumonia of both lungs simultaneously; in the ratio of 7 to 2.

2. The right lung is more frequently the seat of pneumonia than the left one—in the ratio of 2½ to 1. The greater frequency of the disease in the right side had already been noticed by previous writers, although they differ from M. Pelletan, and from each other as to the exact ratio of frequency.

3. The base of the lung is more frequently effected than the summit—in the ratio of 1½ to 1.

4. The age or duration of the pneumonia being the same, the degree or severity of the disease has been found to vary much in different cases; and it is only in the extreme limits that it has been possible to establish a relation between the duration of the inflammation and its development.

(The meaning of this sentence is anything but clear: we scarcely know what the author wishes to announce.)

5. The disease is twice as frequent between 17 and 37 years of age, as at every other period of life.

6. It is more frequent in men than in women;—in the ratio of 10 to 1.

7. The influence of cold as the exciting cause has been remarkable in seven-ninths of all the cases.

8. The frequency of the pulse has not afforded any exact measure or indication of the intensity of the disease nor of the point which it has reached. It is not, however, to be denied, that the acceleration of the pulse is a sign of considerable value, especially when it continues or increases after blood-letting.

9. The frequency of the inspirations has seemed to measure very exactly the degree of the disease, and to indicate its gravity.

10. Prostration and delirium have in general existed when the inflammation occupied the summit of the lung; this coincidence was of much more frequent occurrence than in pneumonia of the base.

11. As to the treatment of pneumonia, when it is *franchement inflammatoire*, repeated blood-letting, according to the formula of M. Bouillaud, formed its basis. There were only two deaths in 55 cases; and the duration of the disease was incontestibly shortened.

12. Blisters were rarely useful in adults; occasionally in children; always in old persons.

M. Rayer, in reporting upon the memoir of M. Pelletan, very properly showed that the question of the statistical or arithmetical history of pneumonia, or of any other disease, although apparently very simple if deduced from an ample number of cases, contains various elements which require to be attended to with the greatest care.

For in truth pneumonia is not the same malady in children as it is in adults and in old persons: and again, when it prevails epidemically, it is in many respects very different from that form which occurs in a plethoric person after exposure to cold. It is necessary also to take account of secondary pneumonias, of bilious complications, and of a multitude of other circumstances, before one can fairly draw any conclusions of general application.—*Medico-Chirurgical Review, from Gazette Medicale.*

*Remark.*—It will be observed by these very brief remarks of M. Rayer that he is a much less hasty reasoner, and less *heroic* practitioner than his confrere, M. Bouillaud.—*Rev.*

---

*Apoplexy of the Lungs, a frequent Cause of Sudden Death.*—Taking the term *apoplexy* as designating an engorged or highly congested state of a part dependent on, or at least associated with, a partial paralysis of its nerves, it would indeed be rather surprising if the lungs were exempt from such a seizure.

. . . . . . . . . "Why may we not presume that there may be a primary lesion of the functions of the pneumo-gastric nerves, inducing an extinction of the contractile and sensitive properties of the lungs, which will then become penetrated with serous or sanguineous fluid, just as the brain and its vascular apparatus are affected in similar circumstances: in truth, such cases are not unfrequent in old people, and in younger persons of a cachectic disposition. It is admitted by all that the encephalon is susceptible of a sudden compression, arising either from a mere turgescence of its vessels, or from a sanguineous effusion in consequence of the rupture of these vessels. In the same manner the substance of the lungs may become, as it were, inundated with an unusual quantity of blood, or this fluid may be extravasated from its vessels into the pulmonary tissue. Examples of both these conditions are not unfrequent in young persons who are of a vigorous and plethoric habit of body, especially after any violent exertion, as boxing, rowing, &c. Recovery from the former condition is not uncommon; the second form usually proves fatal.

*Bartholin*, in his Tractatus de Pulmone, states that, in many cases of sudden death, the cause is attributable to a sudden irruption of blood into the lungs, and a consequent stagnation of it in their soft texture.

The following remarkable instance is recorded by *Bonetus*, and entitled, *Suffocatio a crasso nigroque succo cordis sinus infarcirente ac posteriores lobos pulmonum.*

*Case.*—A man, 50 years of age, and of a robust habit of body, was commencing his breakfast, when his breathing became suddenly oppressed, and he felt as if he should be suffocated. In spite of the efforts of his physician he died about noon, retaining his faculties to the very last.

The body quickly became enormously emphysematous, and of a dark livid colour all over.

On dissection, the abdominal viscera were found in a healthy condition, with the exception of their appearing of a livid hue from a highly gorged state of their vessels. The texture of the lungs, especially in the posterior part of the lobes, was indurated and as black as if it had been soaked in ink. There was no fluid in the pericardium: the right cavities of the heart contained a dark-coloured blood, of a thick consistence but without coagula.

*Dionis* has recorded an interesting case of a similar nature:—

*Case* 2.—On the 16th July, 1691, the Marquis de Louvois, having dined *en bonne compagnie*, went to the council chamber to read a letter to his majesty. While reading he was obliged to stop, and left the palace immediately for his own house. He requested *Dionis* to bleed him immediately, as he felt as if he should be suffocated. His anguish became, nevertheless, still more distressing; and he complained of a peculiar sensation in his belly, as if it would open. In half an hour from the moment of seizure, he was a corpse.

On dissection, the brain was found to be quite normal; the stomach was full of food; the lungs were swollen and distended with blood the heart was flabby, and its texture was of a remarkably soft consistence; not a drop of blood was in any of its cavities.

*Dionis* very properly attributed the death to a stoppage of the circulation; the lungs were filled with blood because it was retained in their parenchyma, and yet there was none in the heart, because none could flow from the lungs into its cavities.

(Most of our readers will doubtless regard this case of sudden death as the result rather of a cardiac than of a pulmonic lesion. The heart, it seems, was in a state of extreme *ramollissement.*)

*Case* 3.—A gentleman, 54 years of age and of a strong habit of body, became affected with deep melancholy, in consequence of a sudden reverse of fortune; he had never suffered from any pulmonary distress.

He became, however, soon after this time, subject to dyspnœa, and a sense of suffocation. The attacks of these symptoms were only transitory and occasional. While playing with some friends at *piquet*, he experienced a peculiar distressing feeling in the epigastric region, which obliged him to walk about his room. On sitting down he immediately expired.

The body became quickly livid in different parts.

*Dissection.*—When an incision was made through the integuments of the throat, a quantity of blood flowed out; and, on pressing the abdomen, much frothy blood escaped from a wound in the trachea. On laying open this canal and the divisions of the bronchi, their lining mucous membrane was observed to be very highly injected. The pericardium was red and vascular on its inner surface, and contained a small portion of sanguinolent fluid. The aorta and pulmonary artery, as well as the entire surface of the heart, exhibited a deep red colour; the coronary veins were gorged with dark blood, but little was found within the auricles. The muscular tissue of the heart was much softened, and partially converted into a fatty or adipose substance: at one point there was found a mass of adipocire.

MM. *Legallois* and *Thillaye*, who examined the body, were of opinion that the immediate cause of death in this case was the effusion of blood in the bronchi, which caused a sudden suffocation. The passive dilatation of the left ventricle, which no doubt must have caused death sooner or later, could only have contributed to the fatal issue as far as it determined or favoured such an effusion.

*Case* 4.—The Duc de Fleury, 53 years of age, and of a highly nervous temperament, was recovering very favourably from a fracture of the leg, when one evening, after drinking some almond emulsion, he was most unexpectedly seized with a feeling of extreme exhaustion; in half an hour afterwards he was a corpse. The impression was that he had been poisoned.

With the exception of an engorged state of the veins of the dura mater, and two points of ossification in the folds of the falx, no morbid appearances were discovered in the *encephalon.*

The *lungs* were so completely infiltrated with blood as to resemble a sponge which had been immersed in it. The heart was quite empty. On the stomach, and at different points of the intestines, there were patches of ecchymosis pervading the entire thickness of their parietes. The spleen was very large, of a soft consistence, and contained a quantity of dark fetid blood. The whole of the abdominal venous system was gorged and highly congested.

The preceding cases are sufficient to prove that many cases of sudden death are attributable to apoplexy not of the brain, but of the respiratory organs.

Let us now briefly consider what are the *causes* of such a lesion?

It is well known that, in examining the bodies of animals which have died from tying the nervi vagi, the lungs are always found gorged with blood, and the bronchi are obstructed with a reddish coloured sanies.

Now, as the ligature of these nerves necessarily induces a paralysis of the larynx and of the lungs, it seems highly probable that sudden death, when dependent upon pulmonary apoplexy, is also attributable to the same condition.

. . . . . . . . . "The lungs, losing their contractility and sensibility, become passive, and permit the blood sent to them from the heart—which suffers but little from the interrupted functions of the nerves which it receives from each recurrent—to penetrate their texture; the power of the right ventricle becomes feebler as the lungs are more and more distended, and congested with blood; the glottis allows only a small quantity of air to pass at each act of inspiration, and thus the dyspnœa increases more and more until life is at length extinguished. Under such circumstances, the right cavities of the heart are found almost always much distended, while the left cavities are quite empty."

We may therefore fairly infer that paralysis of the nervi vagi is one, and perhaps the most frequent, of the causes of pulmonary apoplexy. Another cause is probably an increased force of the right ventricle of the heart, whereby a larger quantity of blood is sent, and this, too, with such power upon the lungs, that their tissue becomes oppressed and ultimately engorged.

If, at the same time, there be any obstruction to the return of the blood in the pulmonary veins into the left auricle, this engorgement must necessarily increase, and at length be so great as to give rise to extensive extravasation into the air-cells.

. . . . . . . . . . . . "The signs of the two forms of pulmonary apoplexy, to which we have alluded—viz. that dependent upon a paralysis of the nervi vagi, and that arising from the excessive impulsion of the blood through the pulmonic arteries—present certain differences which may serve to distinguish the one form of the disease from the other. The former is most frequent in advanced life, and at first is usually manifested by a sense of extreme weakness and of oppression; the patient grows pale, totters, and perhaps falls down. Nevertheless, he retains—and this circumstance alone is sufficient to shew that the attack is not of cerebral apoplexy—his intellect, and will tell the by-standers that all his distress is in the chest, and that unless relief is speedily given he must soon die. In perhaps a quarter or half an hour afterwards, a cold icy damp breaks out over all the surface, the pulse from being full and strong becomes rapidly weak and fluttering, and the mind begins to waver.

Most frequently the physician arrives only to witness the dying man, or perhaps his corpse; pronounces the case to be one of *apoplexie foudroyante*, and points to the brain as the seat of the attack.

But how very different is the course of cerebral apoplexy! In it the patient at once, and from the very first moment, loses his intellect as well as sensation and power of motion, and he falls into a state of complete stupor; there is usually some distortion of the mouth, and more or less difficulty of swallowing, and the breathing is almost always slow and stertorous: this state usually lasts from twelve to twenty-four hours, or longer. Surely such a case as this has but few features in common with those exhibited by a sudden lesion of the nervous functions of the lungs."

Independently of the symptoms during life, our author is of opinion that the appearances presented by the corpse, before any dissection takes place, indicate the seat of the mischief.

"The eyes are open and projecting, as we notice in drowned persons; a frothy sanguineous mucus oozes from the mouth in greater or less quantity, as the head is elevated or not; and *the body retains for an unusual length of time its heat*, more especially in the epigastric region. Percussion of the chest gives out almost always a dull sound, and this can be attributed only to some recent lesion of the lungs, since, during the continuance of good health, no complaint had been made of the breathing. These details have nothing in common with those observed in the bodies of those who have died from cerebral apoplexy."

The danger of pulmonary apoplexy, especially in old age, seems to be much greater than that of apoplexy of the brain; life is more quickly extinguished, and remedial measures are altogether more inefficacious. And, indeed, we cannot be surprised at this, when we consider that if there be any paralysis of the nerves of the lungs, those of the heart must necessarily suffer at the same time; whereas in cerebral lesions, the movements of the heart are but little affected, in consequence of its chief nervous supplies being derived from the intercostals.

The experiments of *Le Gallois* have proved that the functions of the lungs will continue, as long as the nervi vagi are uninjured. While this, therefore, is the case, the blood continues to be arterialised, and to make its impression on the brain, as long as this organ is susceptible of receiving and transmitting it. But if the lungs be paralysed, the arterialisation of the blood ceases, and consequently all impression on the brain is soon suspended, and the nervous energy exhausted.

Before closing these remarks, we shall briefly notice two cases, to shew what we consider *threatened attacks* of pulmonary apoplexy in young persons.

*Case.*—A young girl, after violent exertion, was seized with shivering and other symptoms of fever. The breathing became much hurried and very short, and the face was swollen and deeply flushed. The patient was excessively restless, and occasionally expectorated blood; she found relief only by sitting close to an open window: there was also intense headach, dilatation of the pupils, obscurity of vision, short and rattling respiration, and the mouth was every now and then filled with a frothy sanguinolent saliva. The suffocation was imminent. The pulse was small, resisting and hard, (*serré*): but, after a copious venesection which was practised, it became soft and compressible. The dyspnœa and tendency to stupor were also at once relieved; some leeches were then applied on each side of the neck, and next day the patient was nearly well.

*Case.*—A lady who had during her three first pregnancies suffered much from headach, vertigo, pains and sense of heat in the chest, attended with expectoration of blood, became pregnant for the fourth time. Again the same train of symptoms made their appearance; she was bled ten times during her gestation, and all the period kept on a very light diet. By this treatment she went on to her full time, and she was safely delivered of a healthy child.

Dr. *Leveillé* embraces the substance of the preceding observations in the following three conclusions.

1. That apoplexy of the lungs has a perfect analogy with apoplexy of the brain.

2. That it (pulmonary apoplexy) almost always proves rapidly fatal, and generally more so than cerebral apoplexy.

3. That when death takes place in from a few minutes to one or two hours, the cause of it will be usually found in the chest and but seldom in the head.—*Ib. from Revue Medicale.*

*Note.*—It is necessary to state that the memoir, from which the preceding observations are drawn, was read before the Institute of Paris so far back as the year 1815, and consequently before the development of the discoveries of recent pathologists on the influence of the diseases of the lungs, and more especially of the heart, in inducing sudden death. It is, however, not only interesting but really instructive occasionally to refer back to the opinions held by experienced physicians before the torch of auscultation was first applied by *Laennec* to illumine the obscurity which hung round thoracic pathology.

In perusing the preceding observations, the reader will probably remark that it is rather singular that the enlightened author should have so much overlooked the almost invariable coexistence of disease of the heart in cases of pulmonary apoplexy; although in almost every instance examined after death, this organ is described to have been flabby, attenuated, and loaded or even penetrated with fatty matter. The particulars indeed, as to the special lesion of its different cavities and their valves, are not given with sufficient minuteness to satisfy the modern pathologist; but we may fairly conclude that in the majority of the cases, at least in those which occurred in advanced life, the right ventricle was in a state of *ramollissement* and of dilatation. Now under such circumstances the impulsion of the blood along the pulmonary arteries is necessarily weak, irregular and uncertain; hence there arises a congested state of the extreme vessels in different parts of the lungs; and if at the same time there should appear to be any obstruction to the free return of the blood from the pulmonary veins into the left auricle, we cannot be surprised that the blood should escape, either by exudation or by extravasation from rupture in the loose cellular substance, and also, into the air-cells of the lungs, and thus give rise to the disease which has been designated *pulmonary apoplexy.*

Such being the explanation of the mischief, we can at once understand *how* it proves so quickly and often so inevitably fatal.

Not a few cases of asthma, or rather of permanent dyspnœa, in old people, are attributable to this state of things. There is no paralysis of the pulmonary nerves, as imagined by Dr. Leveillé, and no primary disease of the lungs themselves, further than their capillary vessels being more or less congested and therefore weakened: it is not so much a *vital* as a *mechanical* lesion.

Such being the fact, the judicious practitioner will, in the treatment of a case of what he may deem to be one of pulmonary apoplexy, avoid any active or very officious treatment, as the powers of life are so languid that any further depression must endanger the feeble spark of life that remains. On the whole, perhaps, the best practice will be found to be with the one hand to relieve the local congestion by the application of numerous leeches or of the cupping instruments to the chest or back, and with the other hand, and at the same time, to administer frequent doses of ammonia and such diffusible stimuli. Blood-letting from the arm is in many cases inadmissible, at least until some degree of reaction from the primary collapse has taken place: the quantity drawn must depend upon the effects produced upon the general circulation; hence the physician must watch these most attentively while the blood is flowing.

The auscultation of the heart will be found to afford a safer and surer test to guide our practice than the examination of the arterial pulse. The exhibition at the same time of small doses of stimulants, more especially of ammonia and æther, or of hot brandy and water, is not at all inconsistent with moderate depletion. For the powers of the system must be kept up to enable us with safety to relieve the engorged vessels; and the very nicety of the management of such cases consists in the contemporaneous use of stimulating and of depletory measures.

As a matter of course these remarks are applicable chiefly to those cases of pulmonary apoplexy which occur in old people, and are accompanied with, if not absolutely dependent on, a weakened state of the right cavities of the heart. That form of the disease which is apt to occur in young plethoric persons, especially during severe and protracted labour or suffering, as for example during accouchement, requires a much more vigorous and more decided lowering treatment; a large opening must be made in a vein, and the blood be allowed to flow freely and copiously; repeated doses of tartar emetic at the same time, so as to induce nausea, will likewise be most useful.—*Rev.*

---

*Gangrene of the Lung. Recovery.*—H. Mulloy, an Irishman, æt. 34, was admitted into the Charing-Cross Hospital, May 18, under the care of Dr. Chowne. He is of light complexion, short, muscular, and firmly built. A week before his admission he had been subject to cold whilst at work. The exposure to cold was followed by a rigor, which ushered in a severe attack of pneumonia, attended with acute pain and distressing cough. He was freely bled and blistered, and subjected to other antiphlogistic treatment by the medical man under whose care he placed himself. When admitted into the hospital, the inflammatory stage of the attack had subsided; but he still laboured under almost incessant cough, with abundant expectoration, amounting to three or four pints in the twenty-four hours. The countenance was somewhat florid; the pulse ninety, regular, and moderately full; the alvine and other functions natural; the cough was attended with but little pain. He did not suffer much on taking a full inspiration. The expectoration, in addition to being very copious, was sanious, of a brownish-red colour, verging in some parts towards a green. The red colour was so marked, that it was evidently an exudation from blood-vessels. The odour of both the sputa and the breath was excessively fœtid. There was no part of the chest which was entirely dull on percussion, nor could any spot be detected where the respiration appeared to be wholly suspended. Strong mucous râle was audible in most parts of the thorax. The complexion, the state of the pulse, the general strength of the patient, and the tendency to hæmoptysis, were all contra-indicative of the use of such tonics as might have been necessary, had the gangrene been the primary disease, and a consequence of debility, accompanied by extreme depression and typhoid tendency. Acid mixture was prescribed with the pill "saponis cum opio" at bed-time, to diminish the irritability of the lungs, and procure sleep.

22. The expectoration is mixed with blood: the countenance rather florid; pulse rather hard; general state, that of fever. He was bled to eight ounces.

After this the general character of the expectoration continued to improve; the colour was less brown; it had more the appearance of muco-purulent expectoration, and its odour was less offensive.

*June* 13. There was a return of hæmoptysis to-day. He was again bled to eight ounces, and a blister was applied over the scrobiculus cordis, at which part he had pain. At this period, twenty-six days after his admission, the disease had lost the characteristics of gangrene of the lung, and had those of hæmoptysis.

29. He again expectorated blood to-day. He was again bled, and had a large blister applied over the sternum.

*July* 17. Since the last report there has been no hæmoptysis; there has, however, been a considerable preponderance of mucus in the sputa, and it is now wholly free from the colour and odour which were at first so predominant. The quantity has been gradually reduced to a few ounces a-day, and the patient continues improving in strength. The treatment has consisted in the occasional administration of mild aperients. During the entire period of his illness he has been kept perfectly quiet, and has been confined to rice-milk and broth diet.

Dr. Chowne observed of this case, that gangrene of the lung was a disease of comparatively rare occurrence, even in an idiopathic form, but still more rare as a sequel of inflammation. When inflammation and gangrene of the lungs co-existed, the inflammation was, commonly, a consequence of the gangrene—a resource of nature to separate and throw off the gangrenous part. There was, sometimes, and, indeed, not unfrequently, a fœtor of the breath and of the expectoration, the consequence of inflammation alone, and resembling both in kind and degree the fœtor of gangrene. In such cases, however, the sanious brown and greenish appearance was generally wanting. Although the case, from having proceeded favourably, necessarily precluded the possibility of arriving at absolute certainty as to the existence of gangrene, yet, so far as symptoms could be relied on, there was not any room for doubt. It might have been observed, that the case had been attended with less prostration of strength; less general depression and anxiety of countenance; less, indeed, of all the ordinary concomitants of gangrene than usual; but this appeared to have been attributable to the nature of the attack and the stamina of the patient.—*London Lancet.*

---

*On the Preservation of Bodies for the Purposes of Dissection.*—We are informed by Dr. O'Shaugnessy, of Calcutta, that since he has tried the practice of injecting bodies with a solution of arsenic—as recommended by Dr. Tranchina of Palermo, and Dr. Dudley of the United States—the study of anatomy has been pursued with greater ease on the banks of the Ganges than in London or Paris.

Dr. Dujat, of the Ecole Pratique in the latter metropolis, has for two years past adopted this plan, and reports very favourably of it, as affording by far the most efficient means of counteracting putrefaction. He says:

. . . . . . . . . . . . "The brain, after several weeks, was as firm as it usually is in a post-mortem examination; and the pathological lesions of several organs retained all their accustomed appearances. The arm of a subject injected with the arsenical solution last March, (1839,) was three months afterwards in a state of perfect preservation, and subsequently it was allowed to dry, the muscles retaining their usual dark red colour.

Even when putrefaction has commenced, the process will be arrested, and the decayed parts will recover much of their firmness and integrity, besides losing all their offensive odour. This process, therefore, affords a most convenient and effectual means of preserving pathological preparations when the weather is very hot, or when we cannot put them up at the time. In preparing skeletons with their natural ligaments, as well also as the skins of animals, there is no antiseptic application so good as that of the arsenical solution."

Dr. Dujat seems not to have much fear that the employment of arsenic in this way is likely to prove at all poisonous to dissectors. "Indeed I am persuaded," says he, "that it renders the hands less liable to suffer from any wounds or punctures in dissection; for the minute quantity of arsenic that could be absorbed in this way can frighten only the homœopathist; and the experience of several anatomists in Paris, Calcutta, and at Lexington in America, has shown that there is little or no danger."

The strength of the solution used for injection by Dr. D. is considerably weaker than that recommended by Tranchina: it is 125 grammes of the arsenious acid to 1½ kilogrammes (or 1500 grammes) of water. It may be injected into either one of the crural or of the carotid arteries. For ordinary dissections, one injection will be found sufficient; but when our object is to preserve a body for a great length of time, it should be repeated a second or even a third time.

The solution of arsenic is altogether preferable to that of corrosive sublimate, as this latter salt is apt to become decomposed, being converted into the proto-chloruret and ultimately into the metallic state.—*Medico-Chirurgical Review*, *from Journal de Chimie.*

---

*On the Remedial Power of the Lactate of Iron.*—This new preparation of steel has already been extensively tried by some of the leading hospital physicians in Paris, and has met with their unqualified approbation.

The following extracts from a memoir by MM. Gelis and Conté will be sufficient to introduce it to the notice of the English reader.—*Med. Chirur. Rev.*

"Several reasons have induced us to select the combination of the protoxide with the lactic acid: this acid is widely diffused through the economy; there is, perhaps, not one part of the body which does not contain a notable quantity of it. Berzelius has detected it in muscle, in milk, and in all the secretions; the perspirable matter owes its acidity to its presence, and a considerable quantity is found in the urine. The solvent power of the gastic juice is perhaps mainly attributable to the presence of the lactic acid: the traces of the hydrochloric are, it is now generally admitted, very feeble.

It must, therefore, be the lactate that is formed in the stomach, when any steel medicine is swallowed.

It is easily prepared by treating iron filings with diluted lactic acid. The water is decomposed, hydrogen is evolved, and the oxygen combines with the iron. When the evolution of the gas ceases, the solution is filtered and then evaporated until a pellicle forms on the surface: the salt chrystallises on cooling.

The lactate is not very soluble in water, and a high heat decomposes it. It is not readily affected by exposure to the air.

It may be administered in the form of pastilles, drops, or lozenges: the sugar which enters into the composition of these prevents the further oxydation of the salt. The dose is from four to fifteen grains.

The authors adduce several cases of chlorosis and other states of the system which are usually relieved by steel medicines, drawn from the practice of MM. Bouillaud, Rayer, Beau, &c. in which the lactate was administered with excellent effects: in some it succeeded after the usual ferruginous preparations had been fairly used without benefit.

M. Bouillaud has reported most favourably of this new medicine to the Academy: he has used it in 21 cases, and in all it produced excellent effects. It seems to have a marked influence in increasing the appetite. The dose was from six to fifteen pastilles (five centigrammes in each:) perhaps each patient took eight or ten grammes in all.

Professor Fouquier confirmed the favourable report communicated by M. Bouillaud. He stated, as his opinion, that the lactate would become one of the most valuable and standard ferruginous preparations used in medicine.

---

*On the Cause of Milky Blood.*—The experiments of Lecanu, Bertazzi, Christison, and others, have most satisfactorily shown that this abnormal condition of the blood depends upon the presence of an unusual quantity of fatty matter which is suspended in the serum. While in healthy blood the proportion of fatty matter does not exceed from one to two parts in 1,000, it has been found occasionally in cases of milky blood, as high as 30, 40, or even 100 parts.

It is a curious subject of pathological chemistry to explain how this increase of fatty matter in the serum is induced.

It has been stated by some writers that fatty matter may enter the torrent of the circulation in two different ways—either along with the chyle by the thoracic duct, or by the branches of the vena portæ. Schultz found that the blood of this vein contained double the quantity of fatty matter that the venous blood elsewhere did. As a large portion of it is no doubt expended in the secretion of the bile, it must follow that, whenever this secretion is interrupted, a larger proportion than usual will remain uneliminated from the system, and will consequently become diffused over the entire circulating mass.

But independently of this cause of an accumulation of fatty matter in the blood, the same condition of this fluid may very probably arise from a rapid absorption of the subcutaneous fat, or from any cause which may tend to impede its deposition in the cellular tissue.

By attending to these suggestions, we may be able to explain how this morbid alteration of the blood may be observed in diseases which have little or no analogy with each other, and to anticipate, in some measure, the description of cases in which it will be most likely to occur.—*Ib. from Annales de la Societé de Med. de Gand.*

